# Exogenous melatonin enhances salt secretion from salt glands by upregulating the expression of ion transporter and vesicle transport genes in *Limonium bicolor*

**DOI:** 10.1186/s12870-020-02703-x

**Published:** 2020-10-27

**Authors:** Junpeng Li, Fang Yuan, Yanlu Liu, Mingjing Zhang, Yun Liu, Yang Zhao, Baoshan Wang, Min Chen

**Affiliations:** Shandong Provincial Key Laboratory of Plant Stress, College of Life Science, Shandong Normal University, 88 Wenhua East Road, Jinan, 250014 P.R. China

**Keywords:** Ion homeostasis, *Limonium bicolor*, Melatonin, Salt gland, Salt secretion

## Abstract

**Background:**

Salt, a common environmental stress factor, inhibits plant growth and reduces yields. Melatonin is a pleiotropic molecule that regulates plant growth and can alleviate environmental stress in plants. All previous research on this topic has focused on the use of melatonin to improve the relatively low salt tolerance of glycophytes by promoting growth and enhancing antioxidant ability. It is unclear whether exogenous melatonin can increase the salt tolerance of halophytes, particularly recretohalophytes, by enhancing salt secretion from the salt glands.

**Results:**

To examine the mechanisms of melatonin-mediated salt tolerance, we explored the effects of exogenous applications of melatonin on the secretion of salt from the salt glands of *Limonium bicolor* (a kind of recretohalophyte) seedlings and on the expression of associated genes. A pretreatment with 5 μM melatonin significantly improved the growth of *L. bicolor* seedlings under 300 mM NaCl. Furthermore, exogenous melatonin significantly increased the dry weight and endogenous melatonin content of *L. bicolor*. In addition, this treatment reduced the content of Na^+^ and Cl^−^ in leaves, but increased the K^+^ content. Both the salt secretion rate of the salt glands and the expression level of genes encoding ion transporters (*LbHTK1*, *LbSOS1*, *LbPMA*, and *LbNHX1*) and vesicular transport proteins (*LbVAMP721*, *LbVAP27*, and *LbVAMP12*) were significantly increased by exogenous melatonin treatment. These results indicate that melatonin improves the salt tolerance of the recretohalophyte *L. bicolor* via the upregulation of salt secretion by the salt glands.

**Conclusions:**

Our results showed that melatonin can upregulate the expression of genes encoding ion transporters and vesicle transport proteins to enhance salt secretion from the salt glands. Combining the results of the current study with previous research, we formulated a novel mechanism by which melatonin increases salt secretion in *L. bicolor*. Ions in mesophyll cells are transported to the salt glands through ion transporters located at the plasma membrane. After the ions enter the salt glands, they are transported to the collecting chamber adjacent to the secretory pore through vesicle transport and ions transporter and then are secreted from the secretory pore of salt glands, which maintain ionic homeostasis in the cells and alleviate NaCl-induced growth inhibition.

**Supplementary information:**

**Supplementary information** accompanies this paper at 10.1186/s12870-020-02703-x.

## Background

Plants are challenged by various biotic and abiotic stresses throughout their growth and development [1–3]. Salt stress is a ubiquitous abiotic environmental stress that negatively affects plants by inducing first osmotic stress and then ionic stress and oxidative stress, resulting in growth retardation, yield losses, and plant death [4–7].

Salt-tolerant plants called halophytes have evolved a variety of mechanisms that allow them to meet this challenge and limit the adverse effects of salt on their metabolism [8]. One type of halophyte, the recretohalophytes, secrete excess salt from salt glands or salt bladders located mainly on their leaves, thereby ensuring a lower Na^+^ content in their shoots and avoiding the excessive accumulation of ions in their cells, which protects against salt-associated damage [9, 10].

Although salt glands play a key role in the salt tolerance of plants, the mechanism of salt secretion is unclear. Three mechanisms have been proposed to explain this process: an osmotic mechanism [11], exocytosis [12–14], and transfer systems similar to fluid flow in animals [15]. Yuan et al. [9] proposed that membrane-bound translocating proteins such as plasma membrane (PM) H^+^-ATPase (PMA) and the Na^+^/H^+^ antiporter (SOS1, salt overly sensitive) participate in the salt secretion process. PMA produces a H^+^ electrochemical potential gradient that drives the Na^+^/H^+^ antiporter to expel excess Na^+^ from the cells, which could cause newly acquired ions to enter the salt gland [16, 17]. Ding et al. [17] showed that salt secretion from the salt glands of *Limonium bicolor*, a typical recretohalophyte with a 16-cell salt gland, may be an ion-active transport process, in which PMA and SOS1 play a coordinated role. The increased salt secretion function in the recretohalophyte *Avicennia marina* is accompanied by the upregulated expression of genes encoding PMA, SOS1, NHX1 (tonoplast Na^+^/H^+^ antiporter 1), and vacuolar H^+^-ATPase subunit c (VHA-c1) [18]. Tan et al. [19] used an immunoblot analysis to demonstrate that the protein levels of PMA and NHX1 significantly increased as the rate of salt secretion increased. An RNA-sequencing (RNA-seq) study revealed that the candidate genes encoding membrane-bound ion translocating proteins were associated with the salt secretion function of the salt gland in the recretohalophyte *Reaumuria trigyna* [20]. In the recretohalophyte *Mesembryanthemum crystallinum* L., the increased NHX and H^+^-ATPase (V-ATPase) activity of the vacuoles increased the Na^+^ content in the salt bladder [21]. These results indicate that ion transporters play an important role during salt gland secretion.

There is also substantial evidence supporting the role of vesicle transport in salt gland secretion [9]. In *Limonium* Mill., many small transport vesicles were observed in the secretory cells of the salt glands [12, 22, 23], suggesting that these vesicles may participate in salt gland secretion. Thomson and Liu [24] treated *Tamarix aphylla* with rubidium and observed that the resulting electron-dense region was concentrated in small vesicles. A large number of small vesicles are present in the salt glands of *L. bicolor*, typically fusing with the PM [22]. When *L. bicolor* leaves were treated with brefeldin A, a vesicle secretion inhibitor, the secretion rate of the salt glands was severely inhibited [22]. Similarly, Flowers et al. [25] reported results that supported the hypothesis that vesicle transport plays a key role in salt gland secretion, although they also indicated that transporters may be simultaneously involved. Vesicle transport consumes less energy than transport proteins, and ions can accumulate to a high concentration in the vesicles [26]. Recently, LbSYP61 (syntaxin from plants 61, a vesicle transport protein) was shown to directly regulate salt secretion levels from the salt glands of the recretohalophyte *L. bicolor* [26].

Many factors influence salt secretion; for example, both the salt secretion ability and the density of salt glands of *Glaux maritima* L. increased under salt stress [27]. The secretion ability of *L. bicolor* salt glands was promoted by Ca^2+^ and K^+^ under salt stress [17], whereas the stress-induced accumulation of K^+^ in *L. bicolor* salt gland cells (mainly in the nucleus and cytoplasm) increased their salt secretion ability [22]*.* The detailed mechanisms underlying these changes in secretion are not clear, however.

Melatonin (N-acetyl-5-methoxy-tryptamine) was first discovered in plants in 1995 [28, 29], and has since been identified in a wide variety of plant species. Over the past few years, many functions of melatonin have been revealed in different plant tissues and biological processes [30–32]. Melatonin regulates plant growth and development [33–35], and plays an important role in plant resistance to stresses, especially salt stress [31], drought stress [36], cold stress [37], oxidation stress [38, 39], and nutrient deficiencies [40]. The melatonin content increases in plants under stress, improving the plant’s ability to adapt to these challenges [41].

Melatonin-related regulatory mechanisms have been reported in some studies [42], but more research is needed on the regulation and function of melatonin in plant stress tolerance. All previous research on this topic has focused on the use of melatonin to improve the relatively low salt tolerance of glycophytes by promoting growth and enhancing antioxidant ability [7]. It is unclear whether exogenous melatonin can increase the salt tolerance of halophytes, particularly recretohalophytes, by enhancing salt secretion from the salt glands. If exogenous melatonin increases the salt-secreting ability of recretohalophytes, whether it is by regulating the ion transporters and vesicular transport proteins. With its high salt tolerance and ornamental and medicinal value, *L. bicolor* is a typical pioneer plant that grows on saline alkali land and has a wide planting range [9]. In this study, we examined the effects of melatonin on the salt tolerance of *L. bicolor*, in an effort to elucidate the molecular mechanisms involved in its salt secretion.

## Results

### Melatonin alleviates the NaCl-induced growth inhibition of *L. bicolor* seedlings

**Fig. 1:**
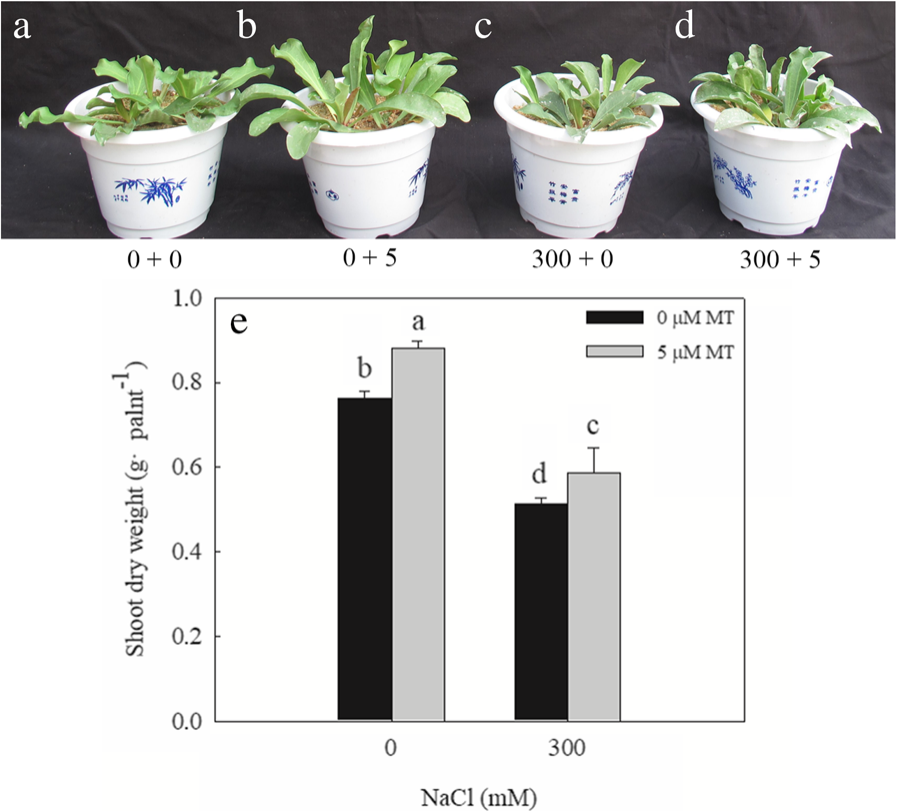
Effects of exogenous melatonin on the growth of *L. bicolor* seedlings under salt stress. **a**-**d**, the *L. bicolor* seedlings, after the six-leaf seedlings with NaCl and melatonin (MT) treatment for 15 consecutive days. **a**, 0 + 0, 0 mM NaCl+ 0 μM MT; **b**, 0 + 5, 0 mM NaCl+ 5 μM MT; **c**, 300 + 0, 300 mM NaCl+ 0 μM MT; *D*, 300 + 5, 300 mM NaCl+ 5 μM MT; **e**, the shoot dry weight of *L. bicolor* seedlings. The values are the average of five biological repeats ± standard deviation. According to Duncan’s multi-range tests, bars that there were significant differences at *P* < 0.05 are labeled with different letters

The growth of *L. bicolor* seedlings was significantly inhibited by a 300 mM NaCl treatment; compared with the control (0 mM NaCl), the salt-treated seedlings had significantly lower dry weights (Fig. 1). Exogenous melatonin (5 μM) significantly promoted seedling growth, with treated seedlings reaching a greater biomass than the controls, both under the 0 or 300 mM NaCl treatments. Exogenous melatonin increased the shoot dry weight of the seedlings exposed to 0 mM NaCl by 17.33%, whereas it increased the dry weight of the salt-treated seedlings by 20.9%.

### Melatonin significantly increases the number of salt glands

**Fig. 2:**
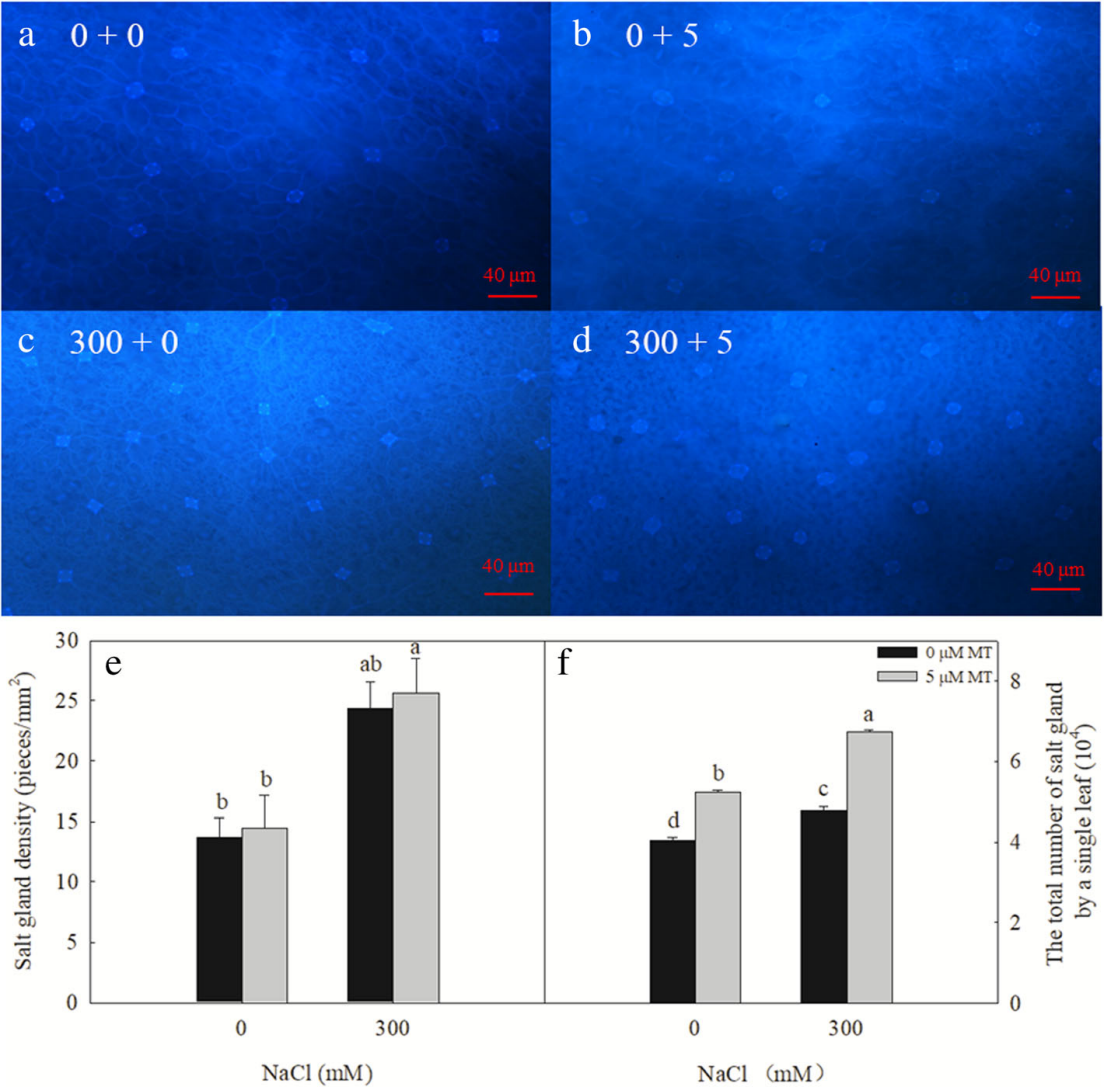
Effects of NaCl and melatonin on the density of salt gland and total number of salt glands of a single leaf. **a**-**d**, the density of salt glands of *L. bicolor* leaves, **a**, 0 + 0, 0 mM NaCl+ 0 μM MT; **b**, 0 + 5, 0 mM NaCl+ 5 μM MT; **c**, 300 + 0, 300 mM NaCl+ 0 μM MT; **d**, 300 + 5, 300 mM NaCl+ 5 μM MT; **e**, the density of salt glands of *L. bicolor* leaves; **f**, total number of salt glands of a single leaf. Bar = 40 μm. Arrows indicate salt glands. The values are the average of five biological repeats ± standard deviation. According to Duncan’s multi-range tests, bars that there were significant differences at *P* < 0.05 are labeled with different letters

The 300 mM NaCl treatment significantly increased the density of salt glands produced on the *L. bicolor* leaves, and the total number of salt glands on a single leaf. The exogenous melatonin treatment had no significant effect on the density of the salt glands on the leaves of plants subjected to either 0 or 300 mM NaCl (Fig. 2a–e). In contrast, the melatonin treatment significantly increased the total number of salt glands produced by each leaf (25.5 and 41.2% increases in the 0- and 300-mM NaCl-treated plants, respectively; Fig. 2f).

### NaCl and exogenous melatonin increase the endogenous content of melatonin in *L. bicolor* leaves

**Fig. 3:**
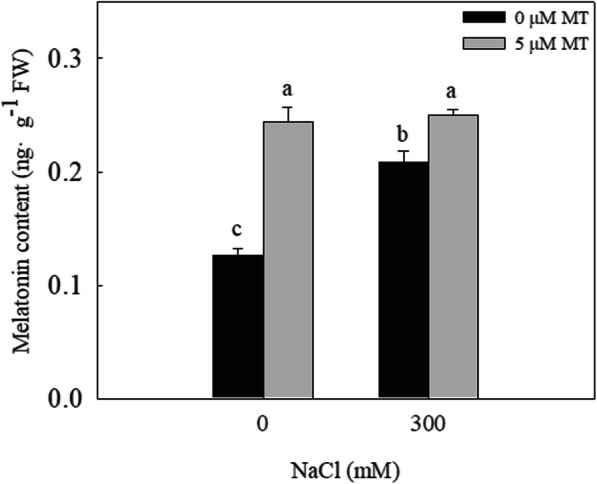
The melatonin content of *L. bicolor* leaves under melatonin and NaCl treatment. The values are the average of five biological repeats ± standard deviation. According to Duncan’s multi-range tests, bars that there were significant differences at *P* < 0.05 are labeled with different letters

The endogenous melatonin content of the *L. bicolor* leaves significantly increased under the 300 mM NaCl treatment (Fig. 3). The exogenous melatonin treatment significantly increased the endogenous melatonin content of the *L. bicolor* leaves, with increases of 97.5% in the 0 mM NaCl-treated plants and 19.1% in the 300 mM NaCl-treated plants.

### Melatonin improves ionic homeostasis under NaCl stress

**Fig. 4:**
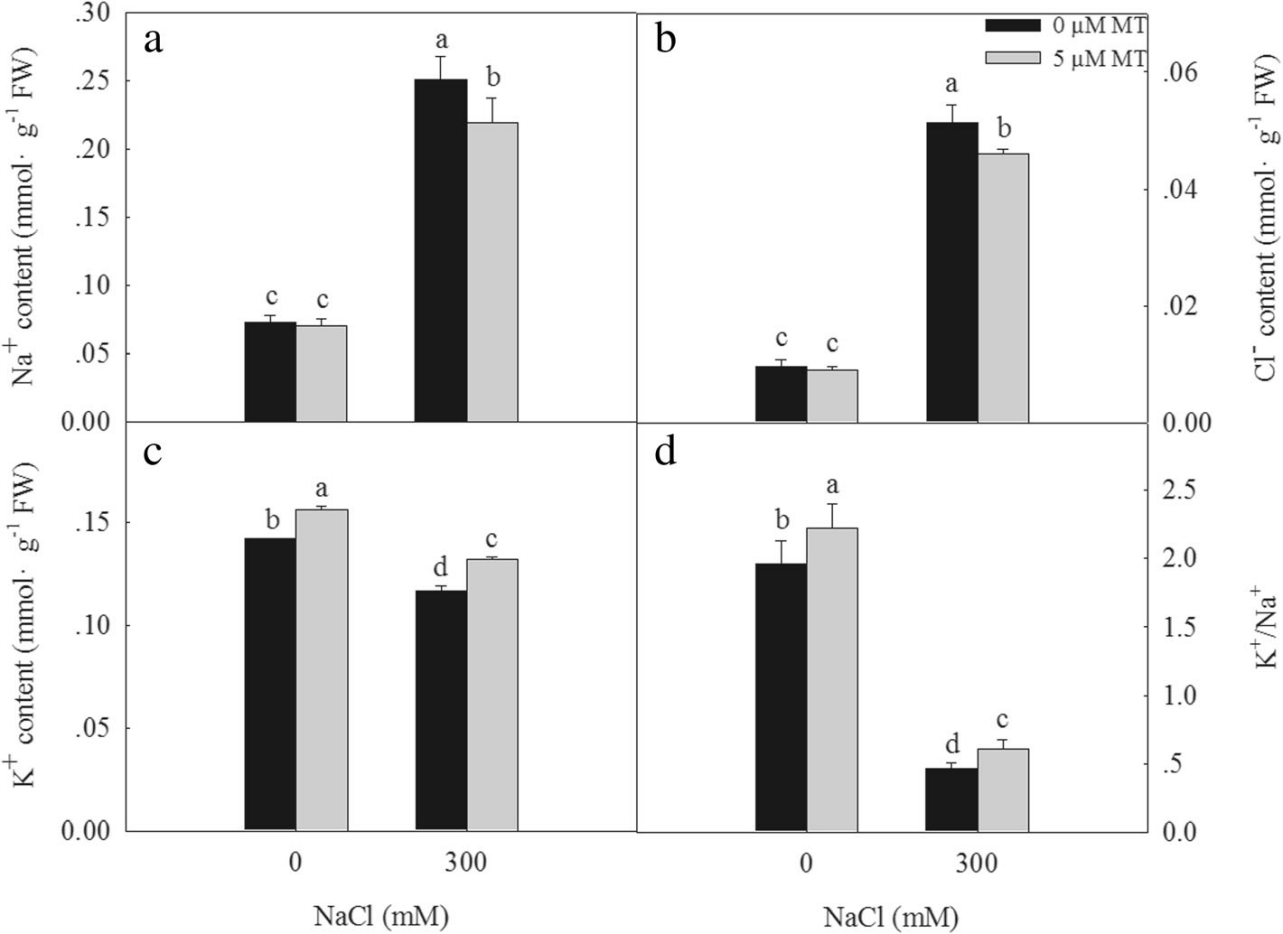
Effects of NaCl and exogenous melatonin on Na^+^ (**a**), Cl^−^ (**b**), K^+^ (**c**) content and K^+^/Na^+^ (**d**) of *L. bicolor* leaves. The values are the average of five biological repeats ± standard deviation. According to Duncan’s multi-range tests, bars that there were significant differences at *P* < 0.05 are labeled with different letters

Salt stress leads to an ionic imbalance in plants by increasing the content of Na^+^ and decreasing the content of K^+^, resulting in the inhibition of plant growth and development, and possibly even plant death. Treatment with 300 mM NaCl significantly increased the Na^+^ (Fig. 4a) and Cl^−^ (Fig. 4b) contents of the *L. bicolor* leaves, while significantly decreasing the K^+^ (Fig. 4c) content and the K^+^/Na^+^ ratio (Fig. 4d). The exogenous melatonin treatment significantly decreased the Na^+^ and Cl^−^ contents of seedlings subjected to the 300 mM NaCl treatment, while significantly increasing the K^+^ content and the K^+^/Na^+^ ratio. These results indicate that melatonin maintains the ion homeostasis of *L. bicolor* leaves under salt stress.

### Melatonin promotes salt secretion from the salt glands

**Fig. 5:**
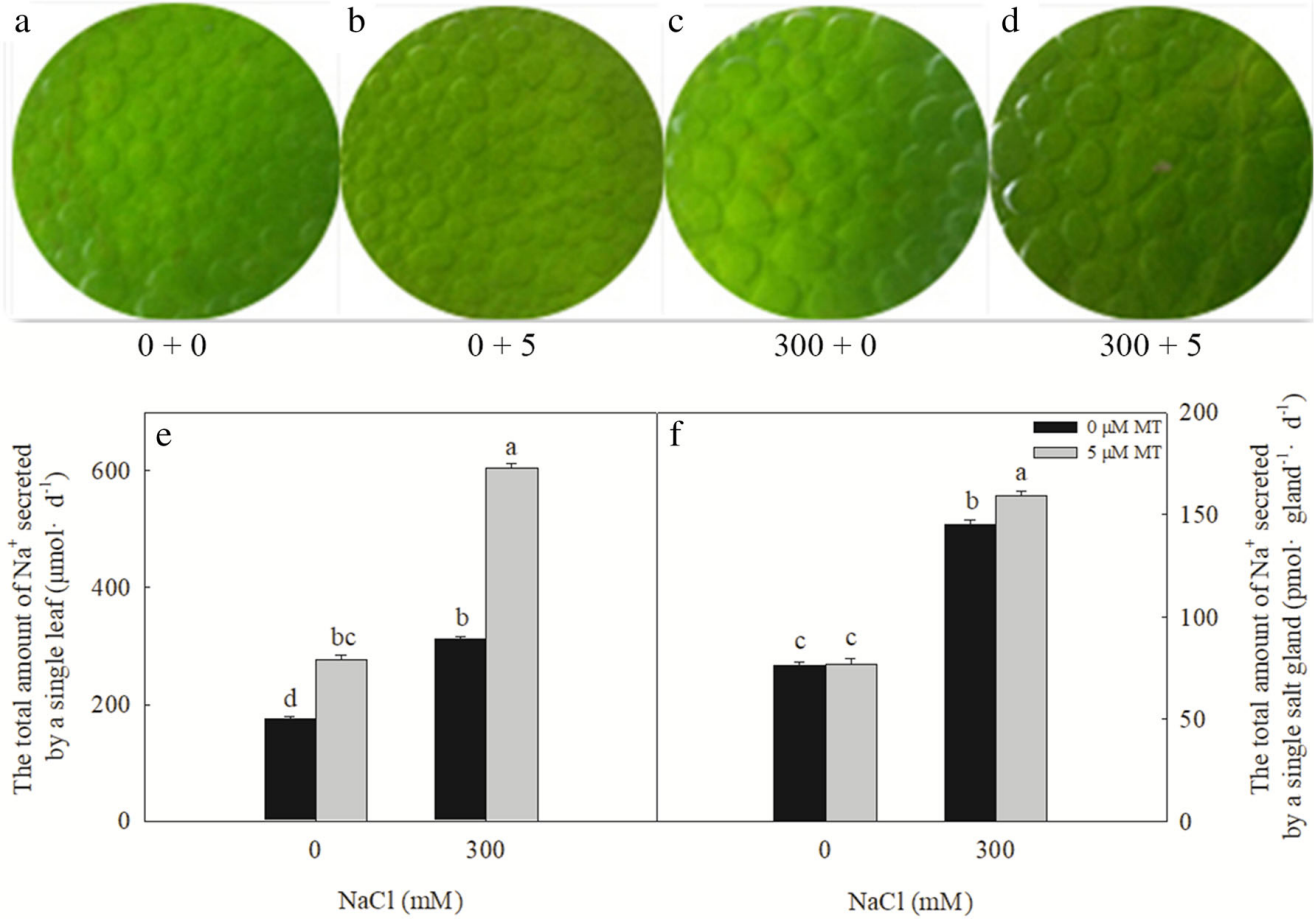
Effects of NaCl and melatonin on salt secretion of salt glands of *L. bicolor* leaves. **a**-**d**, the ability of salt secretion of leaves with leaf disc method, **a**, 0 + 0, 0 mM NaCl+ 0 μM MT; **b**, 0 + 5, 0 mM NaCl+ 5 μM MT; **c**, 300 + 0, 300 mM NaCl+ 0 μM MT; **d**, 300 + 5, 300 mM NaCl+ 5 μM MT; **e**, The total amount of Na^+^ secreted by a single leaf; **f**, the total amount of Na^+^ secreted by a salt gland. The values are the average of five biological repeats ± standard deviation. According to Duncan’s multi-range tests, bars that there were significant differences at *P* < 0.05 are labeled with different letters

*L.bicolor* is a typical recretohalophyte and its growth and development in high-salt environments are closely related to its salt-secretion ability. The 300 mM NaCl treatment significantly increased the amount of salt secreted from a single leaf and a single salt gland (Fig. 5c, e, and f). Salt secretions also significantly increased following the 5 μM melatonin treatment, both in the 0- and 300-mM NaCl-treated plants. Under 0 mM NaCl, 5 μM melatonin had no significant effect on the amount of salt secreted from a single salt gland, although the melatonin treatment significantly increased the amount of salt secreted from a single salt gland in the salt-stressed plants.

### Melatonin upregulates the expression of ion transporter and vesicle transport genes

**Fig. 6:**
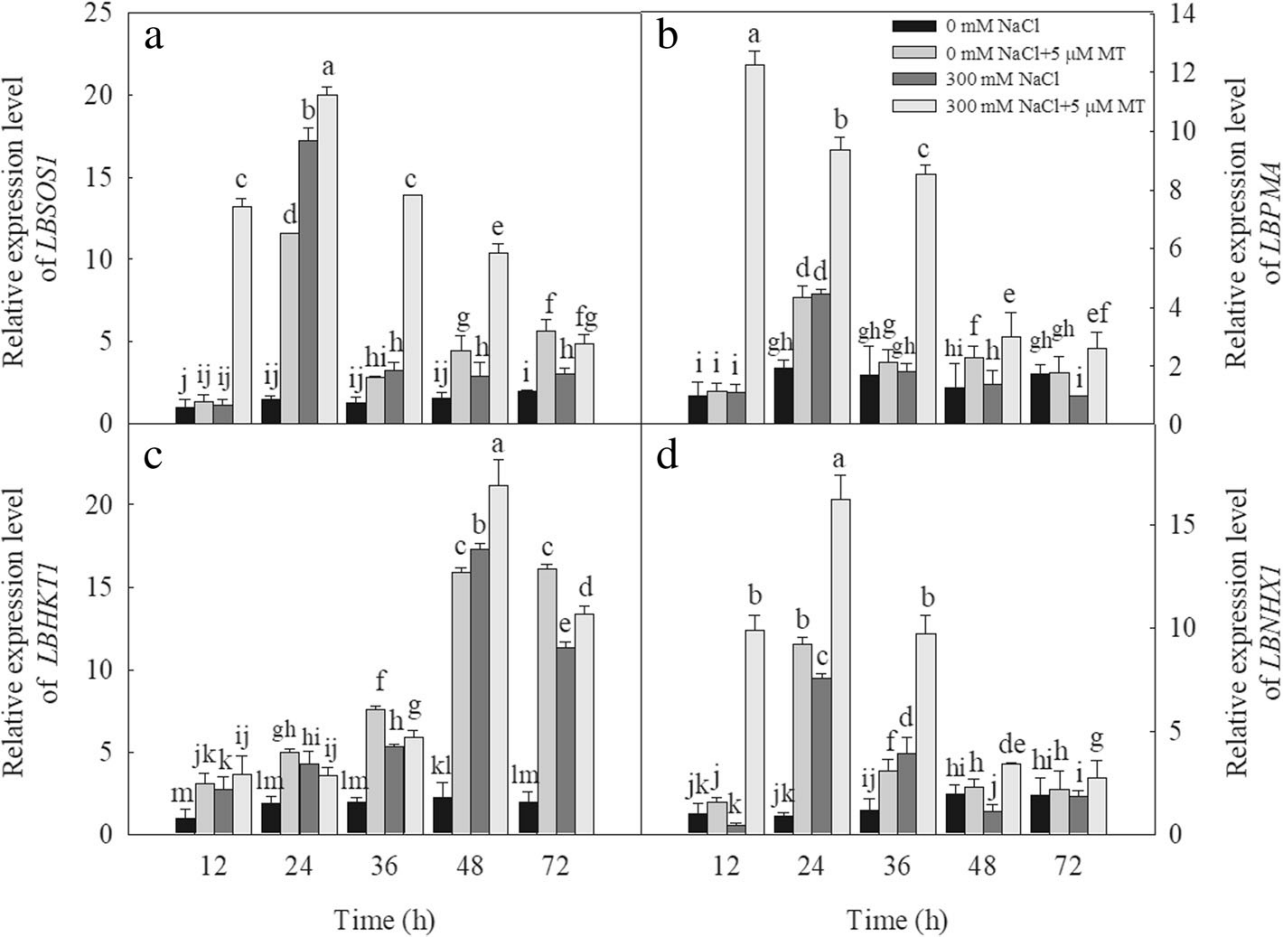
Effects of NaCl and melatonin on ions transporter genes (*LBSOS1*, *LBPMA*, *LBHKT1* and *LBNHX1*) expression in *L. bicolor* leaves in 6-wk-old seedlings at different time points after NaCl (300 mM) and melatonin (5 μM) treatment. (**a**) Transcript level of *LBSOS1* (salt overly sensitive 1), (**b**) Transcript level of *LBPMA* (H^+^-ATPase genes), (**c**) Transcript level of *LBHKT1* (high-affinity K^+^ transporter 1), (**d**) Transcript level of *LBNHX1* (tonoplast Na^+^/H^+^ antiporter). The values are the average of three biological repeats ± standard deviation. According to Duncan’s multi-range tests, bars that there were significant differences at *P* < 0.05 are labeled with different letters

We examined the effect of melatonin treatment on the expression levels of ion transporter-related genes in salt-stressed plants. Following the 300 mM NaCl treatment, the expression levels of *LbSOS1* were significantly upregulated after 24, 36, 48, and 72 h (Fig. 6a); *LbPMA* was upregulated after 24 h (Fig. 6b); *LbHKT1* (high-affinity potassium transporter 1) was upregulated after 12, 24, 36, 48, and 72 h (Fig. 6c); and *LbNHX1* was upregulated after 24 h and 36 h (Fig. 6d), relative to the control. The 5 μM melatonin treatment significantly upregulated the expression levels of *LbSOS1* after 24, 36, 48, and 72 h (Fig. 6a); *LbPMA* after 24 h (Fig. 6b); *LbHKT1* after 12, 24, 36, 48, and 72 h (Fig. 6c); and *LbNHX1* after 24 h and 36 h (Fig. 6d), relative to the control. The combination of the 300 mM NaCl and 5 μM melatonin treatments significantly upregulated the expression levels of *LbSOS1*, *LbPMA*, *LbHKT1*, and *LbNHX1* after 12, 24, 36, 48, and 72 h (Fig. 6).

**Fig. 7:**
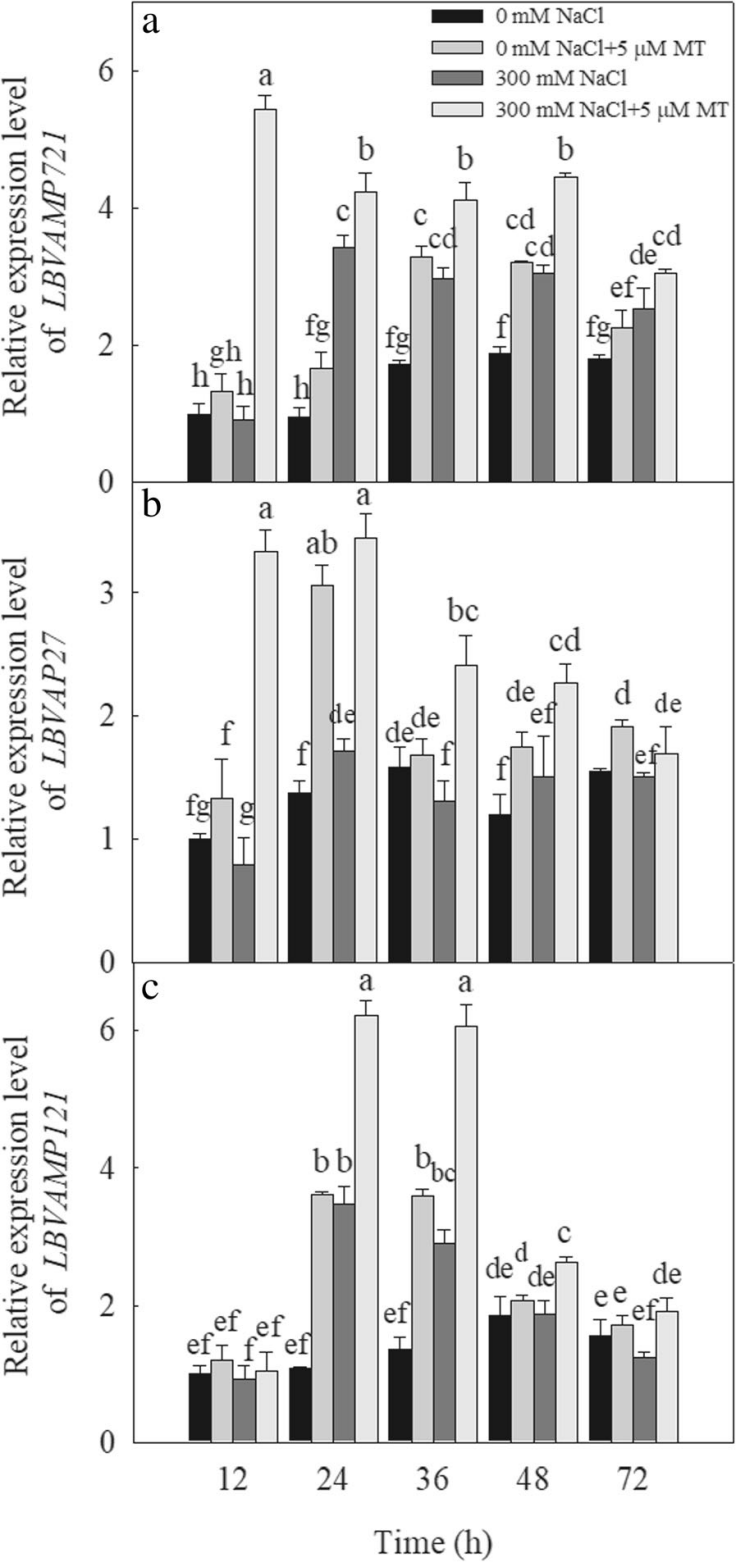
Effects of NaCl and melatonin on vesicle transport related genes *LBVAMP721* (*LB00297*), *LBVAP27* (*LB11044*) and *LBVAMP121* (*LB11277*) expression in *L. bicolor* leaves in 6-wk-old seedlings at different time points after NaCl (300 mM) and melatonin (5 μM) treatment. (**a**) Transcript level of *LBVAMP721* (vesicle-associated membrane protein 721), (**b**) Transcript level of *LBVAP27* (vesicle-associated protein 27), (**c**) Transcript level of *LBVAMP121* (vesicle-associated membrane protein 121). The values are the average of three biological repeats ± standard deviation. According to Duncan’s multi-range tests, bars that there were significant differences at *P* < 0.05 are labeled with different letters

We also measured the expression levels of the vesicle transport-related genes in plants subjected to various salt and melatonin treatments. The 300 mM NaCl treatment significantly upregulated the expression levels of *LbVAMP721* after 24, 36, 48, and 72 h; *LbVAP27* after 24 h; and *LbVAMP121* after 24 and 48 h. The 5 μM melatonin treatment significantly upregulated the expression levels of *LbVAMP721* after 24, 36, and 48 h; *LbVAP27* after 24 h; and *LbVAMP121* after 24 and 36 h. The combination of the 300 mM NaCl and 5 μM melatonin treatments significantly upregulated the expression levels of *LbVAMP721* after 12, 24, 36, 48, and 72 h (Fig. 7a); *LbVAP27* after 12, 24, 36, and 48 h (Fig. 7b); and *LbVAMP121* after 24, 36, and 48 h (Fig. 7c).

## Discussion

Salinity is a wide-ranging environmental stress factor that severely inhibits plant growth and development, reduces crop yield, and even causes plant death [1, 43, 44]. Many plants have developed mechanisms to secrete or tolerate the salts in their cells. The recretohalophyte *L. bicolor* has a strong but poorly understood salt secretion ability, and can therefore be used as a model species to study the development and function of |salt glands [3]. In this study, we revealed that exogenous melatonin significantly increased the ability of the *L. bicolor* salt glands to secrete salt by upregulating the expression of genes encoding ion transporters and vesicle transport proteins, which can increase the salt tolerance of this plant and enable it to grow well in heavily saline soils. This discovery provides a basis for us to further elucidate the salt secretion mechanism of the salt glands in future studies.

Salinity impairs plant growth first by causing osmotic stress, and then ion stress as the salt ions enter the plant body [45–47]. Yuan et al. [3, 23] reported that the growth of *L. bicolor* is inhibited under high-salt conditions (200 mM or more). In the present study, we established that exogenous melatonin application alleviated the inhibition of *L. bicolor* growth caused by treatment with 300 mM NaCl (Fig. 1). This is the first report of this phenomenon in a halophyte. Since even halophytes cannot tolerate large amounts of Na^+^ and Cl^−^ in their cytoplasm, they either compartmentalize excess ions into vacuoles or transport ions into different tissues to maintain cytoplasmic ion homeostasis [1, 48, 49].

As a typical recretohalophyte, *L. bicolor* can excrete excess salts via its salt glands [3, 9], which reduces its Na^+^ and Cl^−^ contents while increasing the K^+^/Na^+^ ratio observed in the leaves (Fig. 4), promoting salt tolerance. The salt secretion rate of the leaves depends on the density and function of the salt glands [10]. Here, we showed that the density of the *L. bicolor* salt glands increased significantly under the independent salt stress, independent melatonin, and combined salt and melatonin treatment (Fig. 2). The increased number and function of salt glands correspondingly enhanced the salt secretion ability of the leaves, which decreased their salt contents and promoted the growth of *L. bicolor* (Figs. 1 and 5).

The secretion rate of the salt glands is affected by many factors [8]. In the present study, exogenous melatonin significantly increased the salt secretion rate of the salt glands and the amount of salt secreted by a single leaf of *L. bicolor* compared with leaves under salt stress alone (Fig. 5). Previous studies have indicated that ion transporters and vesicular transport proteins are involved in salt secretion from the salt glands [12, 17]. In *L. bicolor* leaves, the expression levels of the ion transport-related genes, *LbSOS1*, *LbPMA*, *LbHKT1*, and *LbNHX1*, were upregulated after the application of exogenous melatonin (Fig. 6). SOS1, HKT1, and NHX1 are important ion transport proteins in plants [50]. PMA can provide the driving force for SOS1, and their expression level is positively related to the salt tolerance of plants [50]. Liu et al. [31] showed that exogenous melatonin can upregulate the expression of genes encoding important Na^+^-detoxification transporters under salt stress [31]. Recently, studies have indicated that the exogenous application of melatonin can improve the ion homeostasis of plants under salt stress by upregulating the expression of genes encoding NHX, SOS and other proteins with related functions [30, 51, 52]. Yuan et al. [9] proposed that LbSOS1, LbPMA, and LbHKT1 participate in the salt secretion process, which is consistent with the finding that exogenous melatonin increases the salt secretion rate of the salt glands by upregulating the expression of the ion transport genes. These results indicate that ion transport proteins participate in the salt secretion process in *L. bicolor*.

Ziegler and Lüttge [12] reported that vesicle transport proteins mediate the salt secretion process in the salt glands in the related species *L. vulgare*. Yuan et al. [9] proposed that small vesicles may be involved in transporting salt into and out of the salt glands, which is supported by the results of Lu et al. [26] for *L. bicolor*. We established that the expression levels of the vesicle transport-related genes *LbVAMP721*, *LbVAP27*, and *LbVAMP121* were also upregulated by the melatonin treatment (Fig. 7), which suggested that the vesicle transport proteins can participate in the salt secretion process in this species; however, further research is needed to decipher the mechanism by which exogenous melatonin regulates Na^+^ secretion from the salt gland.

**Fig. 8:**
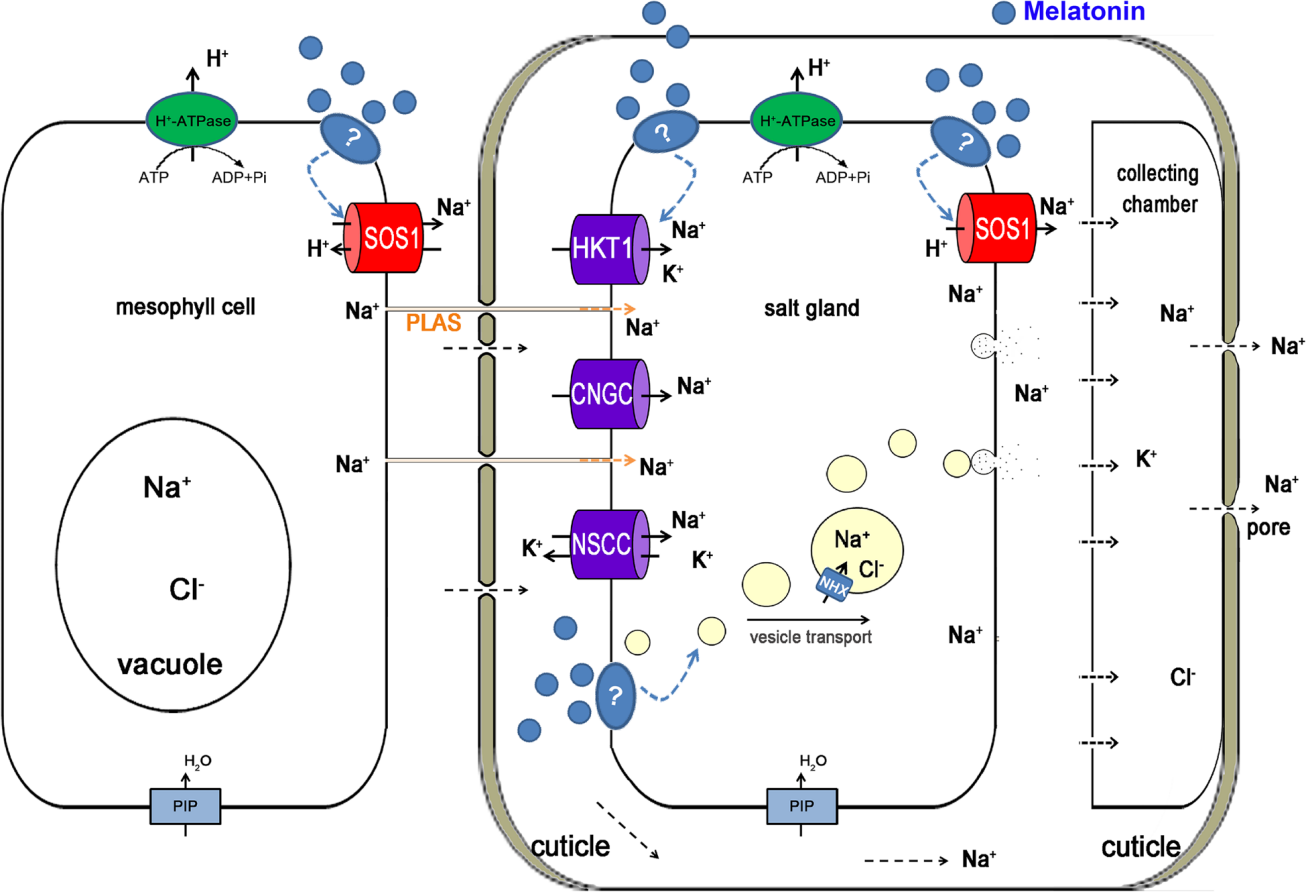
The possible pathway of melatonin increase salt secretion of salt gland (modified on the basis of Yuan et al. 2016). The melatonin up-regulates genes expression related to ions transporters and vesicle transport proteins. The ion transporters transport ions into the salt glands. Ions in the salt glands are transported to the collecting chamber adjacent to the secretory pore through vesicle transport and ions transporter and then are secreted from the secretory pore of salt glands. Blue round indicates melatonin; Green oval located on the plasma membrane indicates LBPMA; Red cylinder located on the plasma membrane indicates LBSOS1; Violet cylinder located on the plasma membrane indicates LBHKT1, LBCNGC (cyclic nucleotide-gated cation channel), LBNSCC (non-selective cationic channel) respectively; Blue rectangle located on the plasma membrane indicates LBPIP (plasma membrane intrinsic protein, aquaporin); Blue cylinder located on the vesicles indicates LBNHX

Our results showed that melatonin can upregulate the expression of genes encoding ion transporters and vesicle transport proteins to enhance salt secretion from the salt glands. Combining the results of the current study with previous research, we formulated a novel mechanism by which melatonin increases salt secretion in *L. bicolor* (Fig. 8). Melatonin upregulates the expression of genes encoding ion transporters and vesicle transport proteins. The ion transporters transport ions into the salt glands. Ions in the salt glands are transported to the collecting chamber adjacent to the secretory pore through vesicle transport and ions transporter and then are secreted from the secretory pore of salt glands, which maintain ionic homeostasis in the cells and alleviate NaCl-induced growth inhibition.

## Methods

### Plant materials and growth conditions

*L. bicolor* seeds were kindly provided by Professor Xu Hualing, Dongying Academy of Agricultural Sciences, Shandong Province. The seeds were sterilized in 0.5% (w/v) sodium hypochlorite solution for 15 min and then cleaned with sterile-distilled water. The seeds were sown on well-washed river sand in plastic pots (16 cm in diameter; after the leaves emerged, the plants were watered with Hoagland’s nutrient solution), which were placed in a growth chamber with 600 μmol m^− 2^ s^− 1^ light (15-h day/9-h night photoperiod), a temperature of 25 ± 3 °C/22 ± 3 °C (day/night), and a relative humidity of 60/80% (day/night).

### Combined NaCl and melatonin treatment

When the seedlings reached the six-leaf stage, they were subjected to NaCl and melatonin treatments. For the NaCl treatment, the seedlings were treated with Hoagland’s nutrient solution containing NaCl, which was increased by 50 mM every 12 h to a final concentration of 300 mM to avoid salt shock. When the NaCl concentration reached 300 mM, melatonin treatment starts, which will treat for 15 days. The control seedlings were treated with Hoagland’s nutrient solution only. To research the effects of melatonin on salt tolerance in *L. bicolor*, the NaCl-treated (the NaCl concentration gradually increased to 300 mM) and control seedlings were irrigated with 0 or 5 μM melatonin (based on the pre-test with various concentrations of melatonin, Fig. S1), which dissolves in the above Hoagland’s nutrient solution. The *L. bicolor* seedlings were treated with various combinations of salt and melatonin every 12 h for 15 consecutive days. Five replicates (3 plants per replicate) were performed for each treatment. After 15 days, the leaves were collected to determine the biological indicators.

### Physiological index measurements

The dry weights of seedlings after 15 days of the treatments were measured as described by Yuan et al. [10]

### Melatonin quantification

The melatonin content of *L. bicolor* leaves was quantified as described by Sun et al. [53]. Briefly, 0.3 g *L. bicolor* leaves were ground into powder in liquid nitrogen, mixed well with 1.5 mL methanol, and incubated overnight at 4 °C. The solutions were centrifuged for 10 min at 10,000×*g* at 4 °C, after which the supernatant was transferred into new test tubes and the liquid was allowed to evaporate. The remaining residues were dissolved in 0.75 mL methanol. A fluorescence detector system (L3000; Rigol Technologies, Beijing, China) was used to determine the melatonin concentration by the area of the peaks identified during high-performance liquid chromatography.

### Ion content measurement

Leaf samples (5 g) were placed into test tubes containing 10 mL ddH_2_O and the tubes were placed in a boiling water bath for 3 h, after which the samples were filtered through filter paper. The supernatant was made up to a volume of 25 mL with ddH_2_O. The Na^+^ and K^+^ contents were measured using a flame spectrophotometer (Model 2655–00 Digital Flame Analyzer; Cole-Parmer Instrument Company, Vernon Hills, Illinois, USA), and the Cl^−^ content was measured using an ion chromatograph (ICS-1100 ion chromatograph; Thermo Fisher Scientific, Waltham, MA, USA), as described by Lin et al. [54]

### Characterization of the *L. bicolor* salt glands

*L. bicolor* leaves (Fig. S2) were cleared using Carnoy’s solution (mixed solution of ethanol and acetic acid (3:1, v/v)), as described by Kuwabara and Nagata [55](2016), after which they were fixed onto microscope slides using Hoyer’s solution [56](Yuan et al., 2014). The diameters and densities of the salt glands were determined using a Nikon fluorescence microscope (ECLIPSE 80i; Nikon, Tokyo, Japan) at × 200 and × 100 magnifications with a standard DAPI filter set under UV excitation (330–380 nm). Digital images were taken using a charge-coupled device camera. Fifteen leaves were measured per treatment, with the measurements taken at the same position on each leaf. The salt glands were counted for a given leaf area to calculate the salt gland density on the abaxial surfaces of the leaves [17]. The total number of salt glands is equal to the salt gland density multiplied by the leaf area. The salt secretion levels under different NaCl and melatonin treatments were determined using the leaf disk method, as described by Lu et al. [26].

### qRT-PCR analysis

The nucleotide sequences of ion homeostasis (*LbHTK1*, *LbSOS1*, *LbPMA*, and *LbNHX1*) and vesicle transport (*LbVAMP721* (vesicle-associated membrane protein 721), *LbVAP27* (syntaxin from plants 27)*,* and *LbVAMP121* (vesicle-associated membrane protein 721)) genes in *L. bicolor* were obtained according to the second-and third-generation RNA sequences [3]. A BLAST search for homologous genes was carried out in both *L. bicolor* and other species, and homologous sequences were downloaded. Primers were designed for cloning the conserved region sequences (800 bp). The conserved region sequences of *LbHTK1*, *LbSOS1*, *LbPMA*, *LbNHX1*, *LbVAMP721*, *LbVAP27*, and *LbVAMP121* were obtained. Beacon Designer (Premier Biosoft, Palo Alto, California, USA) was used to design primers for these seven genes (Table S1). AceQ Universal SYBR Green qPCR Master Mix (Vazyme Biotech, Nanjing, China) and a real-time quantitative PCR instrument (Bio-Rad Laboratories, Hercules, California, USA) were used to perform the real-time PCR. The relative expression of each gene was calculated using the 2^–△△Ct^ method [17, 57], with the housekeeping gene *LbTUBULIN* used as an internal reference.

### Statistical analysis

The statistical analysis was performed using the SPSS software package (version 19.0; IBM, Armonk, New York, USA). The statistical significance was determined using an analysis of variance (ANOVA), and significant differences (*P* < 0.05) between the values were determined using Duncan’s multiple range test.

## Supplementary information

**Additional file 1: Figure S1.** Pre-test with various concentrations of melatonin and found that for the species, 5 μM melatonin significantly improved the growth under control and NaCl treatment.

**Additional file 2: Figure S2.** Leaves for analyzing characterization of the *L. bicolor* salt glands.

**Additional file 3: Table S1.** Primers of candidate genes used for real-time qPCR analysis.

**Additional file 4.** All data generated during this study.

## Data Availability

Not applicable.

## Abbreviations

MT: melatonin
PM: plasma membrane
PMA: plasma membrane H^+^-ATPase
SOS1: salt overly sensitive
NHX1: tonoplast Na^+^/H^+^ antiporter 1
HKT1: high-affinity potassium transporter 1
VAMP721: vesicle-associated membrane protein 721
VAP27: syntaxin from plants 27
VAMP121: vesicle-associated membrane protein 721
