# Supplementary figures and images for: Exogenous melatonin enhances salt secretion from salt glands by upregulating the expression of ion transporter and vesicle transport genes in Limonium bicolor
Source: BMC Plant Biol. 2020 Oct 27;20:493. doi: 10.1186/s12870-020-02703-x (PMC7590734; doi:10.1186/s12870-020-02703-x)

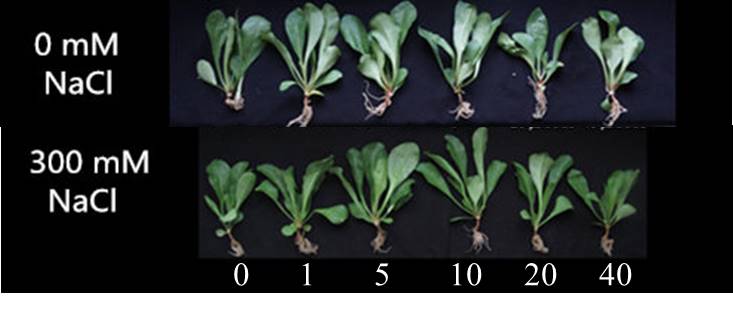

Supplement: Supplementary file 1 — Additional file 1: Figure S1. Pre-test with various concentrations of melatonin and found that for the species, 5 μM melatonin significantly improved the growth under control and NaCl treatment. [file 12870_2020_2703_MOESM1_ESM.jpg]

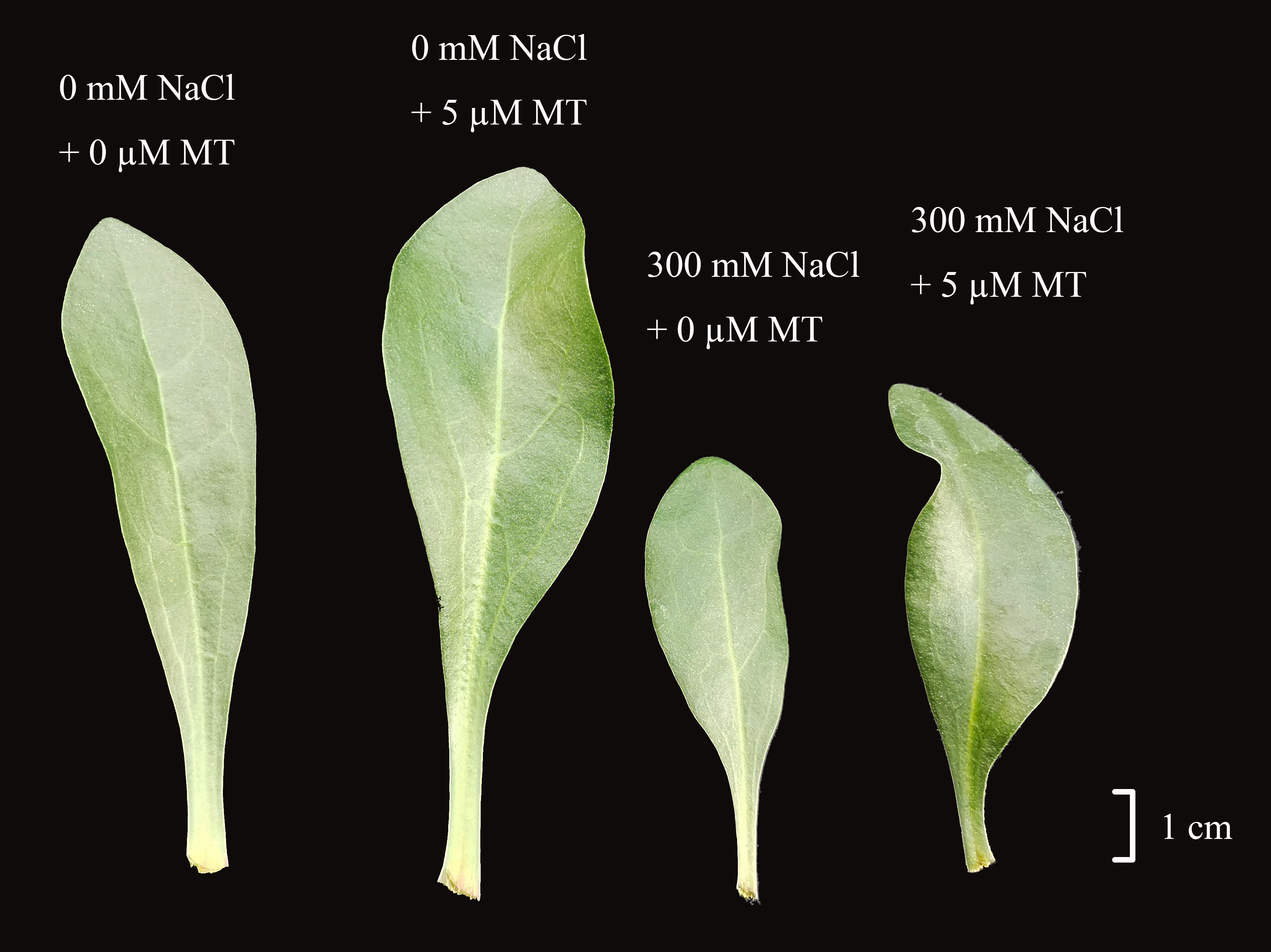

Supplement: Supplementary file 2 — Additional file 2: Figure S2. Leaves for analyzing characterization of the L. bicolor salt glands. [file 12870_2020_2703_MOESM2_ESM.jpg]
