# Supplementary material for: Exogenous melatonin enhances salt secretion from salt glands by upregulating the expression of ion transporter and vesicle transport genes in Limonium bicolor
Source: BMC Plant Biol. 2020 Oct 27;20:493. doi: 10.1186/s12870-020-02703-x (PMC7590734; doi:10.1186/s12870-020-02703-x)
Supplement: Supplementary file 3 — Additional file 3: Table S1. Primers of candidate genes used for real-time qPCR analysis. [file 12870_2020_2703_MOESM3_ESM.docx]

**Table S1.** Primers of candidate genes used for real-time qPCR analysis.

| **Gene ID** | **F1 (5'**-**3')** | **F2 (5'**-**3')** |
| --- | --- | --- |
| *TUBULIN*  *LBHKT1*  *LBSOS1* | GGTTGAGTGAGCAGTTCAC  CGTCGTTATCACTGCTCAG  GTATCTCGCACCGTAGTT | GATAACCAGCCACACCTTAC  GATGTGGTACTGCTGGAGAT  CTCTTAGCCCTGACCAAA |
| *LBPMA* | CTCTTAGCCCTGACCAAA | CCGTCTTCTCAAGTGTTG |
| *LBNHX1*  *LBVAMP721*  *LBVAP27*  *LBVAMP121* | GATCAACGAGTCCATCAC  CGTGGTGAGAAGATTGAG  TCTGGAGAGGACAAGACT  TGAGTTGAGGAAGCAGAC | GCCAGTCACCAGTAGTAT  CCGATGTTCCTTGTGTTC  AAGTTTCTCGCCCTGTTC  GGACGAACGCAATACTTC |
